# High-Efficiency Flicker-Free LED Driver with Soft-Switching Feature

**DOI:** 10.3390/mi13050797

**Published:** 2022-05-20

**Authors:** Hung-Liang Cheng, Lain-Chyr Hwang, Heidi H. Chang, Qi-You Wang, Chun-An Cheng

**Affiliations:** 1 Department of Electrical Engineering, I-Shou University, Kaohsiung 84001, Taiwan; hlcheng@isu.edu.tw (H.-L.C.); lain@isu.edu.tw (L.-C.H.); isu10901014m@cloud.isu.edu.tw (Q.-Y.W.); 2 Department of International Media and Entertainment Management, I-Shou University, Kaohsiung 84001, Taiwan; heidichang@isu.edu.tw

**Keywords:** buck converter, interleaved operation, light-emitting diode (LED), zero-current switching off (ZCS), zero-voltage switching on (ZVS)

## Abstract

A novel interleaved DC-DC buck converter is proposed to drive high-brightness light-emitting diodes (LEDs). The circuit configuration mainly consists of two buck converters, which are connected in parallel and use interleaved operation. Through interleaved operation, the power capability of the converter is doubled. Traditionally, two individual inductors are used in the two buck converters. The difference between conventional parallel-operated buck converters using two energy storage inductors and the proposed circuit is that the proposed circuit uses two small inductors and a coupled inductor that replace the two inductors of the buck converters. In this way, both buck converters can be designed to operate in discontinuous-current mode (DCM), even if the magnetizing inductance of the coupled inductor is large. Therefore, the freewheeling diodes can achieve zero-current switching off (ZCS). Applying the principle of conservation of magnetic flux, the magnetizing current is converted between the two windings of the coupled inductor. Because nearly constant magnetizing current continuously flows into the output, the output voltage ripple can be effectively reduced without the use of large-value electrolytic capacitors. In addition, each winding current can drop from positive to negative, and this reverse current can discharge the parasitic capacitor of the active switch to zero volts. In this way, the active switches can operate at zero-voltage switching on (ZVS), leading to low switching losses. A 180 W prototype LED driver was built and tested. Our experimental results show satisfactory performance.

## 1. Introduction

Compared with fluorescent lamps or high-intensity gas-discharged lamps, light-emitting diodes (LED) have the advantages of higher luminous efficiency, longer lifespans, and higher color rendering, and have been widely used in various lighting systems [1,2,3,4]. In advanced countries, lighting electricity accounts for a high proportion of overall electricity consumption, so research on high-efficiency LED drivers has become an important topic in academia and industry. In general, circuit efficiency and lifetime are two important considerations when designing LED drivers [5,6,7].

The characteristics of LEDs are very similar to those of ordinary diodes, and they are both driven by DC voltage. Researchers have proposed various DC-DC converters to drive LEDs. Because the rated voltage of each LED is very small and increasing the output voltage by connecting many LEDs in series is not a practical approach, buck converters that can easily be pulsed-width modulation (PWM)-controlled to achieve low output voltage are commonly used [8,9,10,11,12]. In addition, the buck converter has a circuit topology in which the energy-storage inductor is connected in series with the output terminal. By designing a buck converter that operates in continuous-current mode (CCM), the inductor can continue to provide current to the output terminal. Compared with other commonly used PWM converters such as boost, buck-boost, flyback converters, etc., the buck converter can use its smaller capacitance to effectively reduce the output voltage ripple to a reasonable value. This means that the buck converter does not require a large electrolytic capacitor that has a low lifespan of usually less than 10,000 h and is usually the bottleneck in the product life of power converters [1,3,13].

Similar to other PWM converters, buck converters have the advantages of simple circuit structure and easy control, yet also have the disadvantage of hard switching; the active switch cannot meet zero-voltage switching on (ZVS), resulting in huge switching losses. In order to solve the problem of hard switching, some soft-switching technologies use active clamping circuits or snubber circuits to enable the active switches to achieve ZVS [14,15,16,17,18]. However, in addition to more complex control, these soft-switching technologies require components such as auxiliary switches, diodes, inductors, and capacitors, resulting in higher product costs. Moreover, the current loop in the active clamp circuit or the snubber circuit also generates conduction loss, and some auxiliary switches cannot meet the ZVS, resulting in high switching losses. Another technique used to make the active switch operate at ZVS is using the control method of synchronous rectification (SR) [19,20,21]. When applying SR technology, MOSFETs must be used to replace the original flywheel diodes in the circuit. In order to ensure ZVS operation, SR technology must detect the inductor current and turn on the active switch when the inductor current drops to a negative value. Therefore, the control circuit of SR technology is more complex due to the need for additional MOSFETs and current detection circuits.

Another major factor affecting converter efficiency is that the power diodes do not operate at zero-current switching off (ZCS). As mentioned before, in order to reduce the current/voltage ripple, the buck converter should operate in CCM so its freewheeling diode is turned off when there is forward current. When a diode does not turn off at zero current due to the rapid return of minority carriers across the space charge region, a large reverse current is generated, which causes problems such as increased diode loss, reduced circuit efficiency, and increased electromagnetic interference (EMI).

Aiming for high circuit efficiency and long product lifetime, we developed a new converter to drive high-brightness LEDs. The circuit topology is mainly composed of two interleaved buck converters in which a coupled inductor is used. Through interleaved operation, the magnetizing current of the coupled inductor continuously flows into the output terminal. The output voltage/current ripple can be effectively reduced without using a large electrolytic capacitor. In addition, the active switches can operate at ZVS, and the freewheel diodes can operate at ZCS. A 180 W prototype LED driver was built and measured to verify the feasibility of this proposed circuit.

## 2. Proposed Circuit Topology and Operating Principle

### 2.1. Circuit Topology

**Figure 1:**
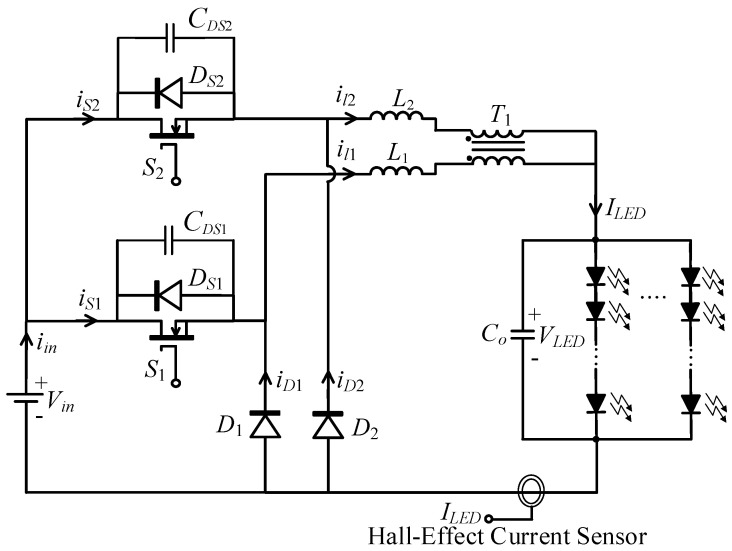
Circuit topology of the proposed LED driver.

**Figure 2:**
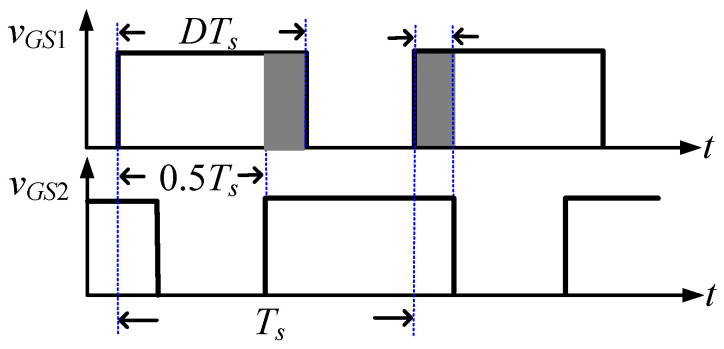
High-frequency waveforms of gated voltages *v*_g_*_S_*_1_ and *v*_g_*_S_*_2_.

Figure 1 shows the proposed LED driver, which is mainly composed of two buck converters. One buck converter consists of a MOSFET *S*_1_, a freewheel diode *D*_1_, an inductor *L*_1_, and one winding of a coupled inductor *T*_1_, while the other converter consists of a MOSFET *S*_2_, a freewheel diode *D*_2_, an inductor *L*_2_, and the other winding of the coupled inductor. Here, the two windings are called primary and secondary windings. Capacitances *C_DS_*_1_ and *C_DS_*_2_ and diodes *D_S_*_1_ and *D_S_*_2_ are the parasitic capacitances and intrinsic diodes of *S*_1_ and *S*_2_. *S*_1_ and *S*_2_ are alternately turned on and off by two gated voltages, *v_G__S_*_1_ and *v_G__S_*_2_. As shown in Figure 2, *v_G__S_*_1_ and *v_G__S_*_2_ are high-frequency square waveforms. They have the same frequency *f_s_* and the same duty cycle *D*, and there is an overlap time during which both square waves are high voltage.

**Figure 3:**
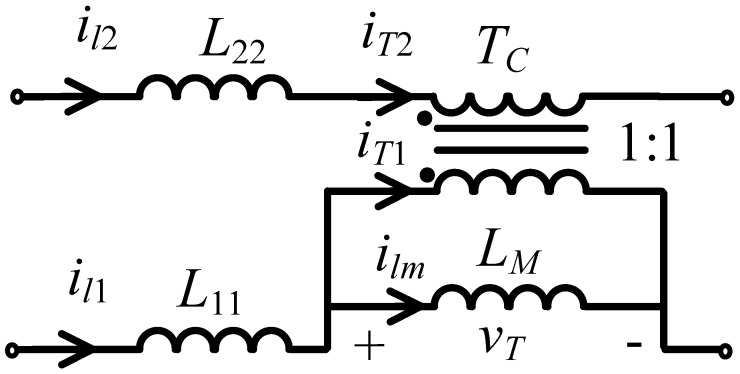
Simplified transformer model of the coupled inductor.

The coupled inductor consists of a magnetic core and two windings with the same number of turns. It is modeled by using a simplified transformer model, as shown in Figure 3, where *L*_1__1_ and *L*_22_ represent the leakage inductance of each winding, *T_c_* is an ideal transformer with a turn ratio equal to 1:1, and magnetizing inductance is *L_M_*. The voltage across *L_M_*, the current flowing in the two windings, and the magnetizing inductance are denoted as $v_{T}$, $i_{T1}$, $i_{T2}$, and $i_{lm}$, respectively. Based on the simplified model, the relation between these currents can be expressed as:

(1) $$i_{l1}=i_{lm}+i_{T1}$$

(2) $$i_{l2}=i_{T2}=-i_{T1}$$

Adding (1) and (2), we obtain:

(3) $$i_{lm}=i_{l1}+i_{l2}$$

Equation (3) states that magnetizing current is equal to the sum of the two winding currents, i.e., if the magnetizing current remains constant, the magnetizing current can be converted to flow between the two windings. Using (3) and Faraday’s law of inductance, the following equation is written:

(4) $$v_{T}=\frac{L_{M}{di}_{lm}}{dt}=\frac{L_{M}d(i_{l1}+i_{l2})}{dt}\;$$

### 2.2. Steady-State Circuit Analysis

#### 2.2.1. Equivalent Circuits of Different Operating Modes

**Figure 4:**
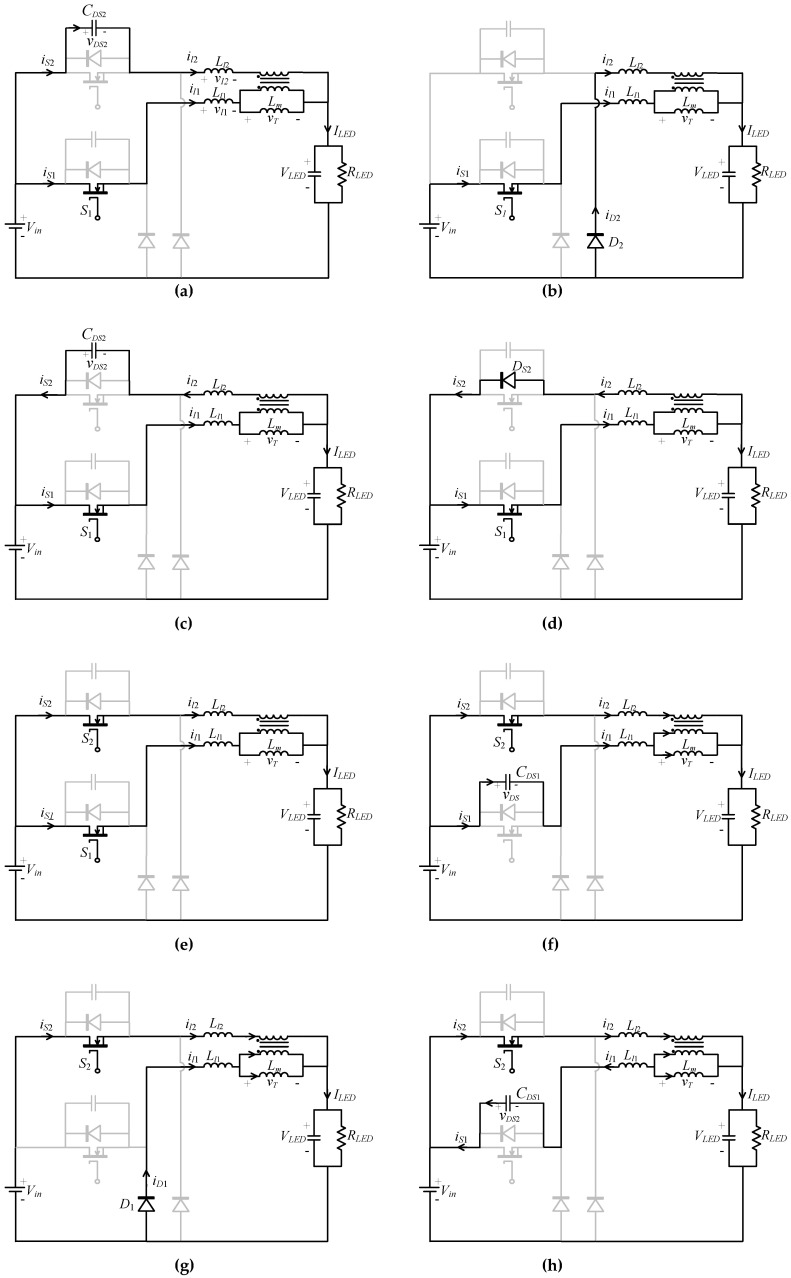

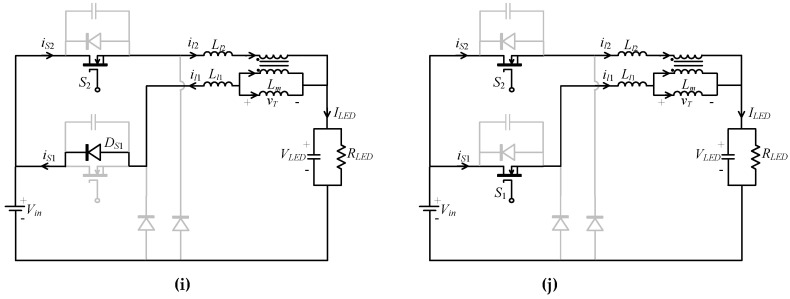
Equivalent circuits of different operating modes. (**a**) Mode I; (**b**) mode II; (**c**) mode III; (**d**) mode IV; (**e**) mode V; (**f**) mode VI; (**g**) mode VII; (**h**) mode VIII; (**i**) mode IX; (**j**) mode X.

In order to ensure ZVS and ZCS operation, buck converters are designed to operate in DCM. At steady-state operation, the circuit operation can be divided into ten operating modes in one high-frequency cycle. Figure 4 shows the equivalent circuits for different operating modes, where the leakage inductance *L*_1__1_ and *L*_22_ are combined with *L*_1_ and *L*_2_:

(5) $$L_{l1}=L_{1}+L_{11}$$

(6) $$L_{l2}=L_{2}+L_{22}$$

As shown in Figure 4a, the voltages across *L_l_*_1_ and *L_l_*_2_ are denoted as $v_{l1}$ and $v_{l2}$, respectively. The following assumptions are made for simplifying the circuit analysis:

1. The resistances of all components are neglected.
2. *L_l_*_1_ and *L_l_*_2_ are equal (*L_l_*_1_ = *L_l_*_2_ *= L_l_*).
3. *L_M_* is large; hence, $i_{lm}$ only changes slightly within one switching period.
4. The output capacitance *C_o_* is large enough to have a constant output voltage $V_{LED}$.

**Figure 5:**
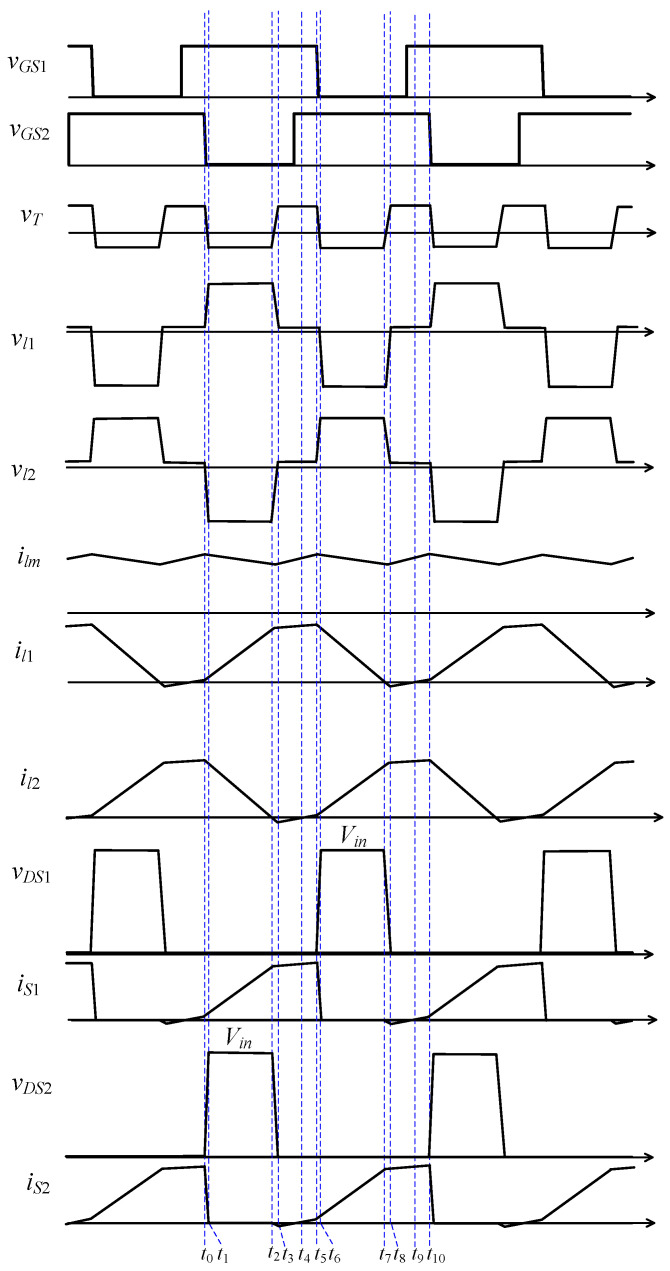
Conceptual waveforms of the main components.

The conceptual voltage and current waveforms of the main components for each operation mode are shown in Figure 5.

#### 2.2.2. Detailed Circuit Analysis of Different Operating Modes

##### Mode I (*t*_0_ *< t < t*_1_)

Before mode I, *S*_1_ and *S*_2_ are both on, as shown in Figure 4j. Because *S*_2_ is turned on before *S*_1_, most of $i_{lm}$ flows through the secondary winding. Therefore, $i_{l2}$ is much larger than $i_{l1}$. Mode I starts at the moment when *S*_2_ is turned off. Figure 4a shows the equivalent circuit. *S*_1_ remains on, and $i_{l2}$ charges the parasitic capacitance *C_DS_*_2_. Because *C_DS_*_2_ is very small and $i_{l2}$ is high, *C_DS_*_2_ is quickly charged to *V_in_*. When $v_{DS2}$ is equal to *V_in_*, mode I ends. As mentioned above, because *C_DS_*_2_ is small and the charging current is large, the duration of this mode is very short.

##### Mode II (*t*_1_ *< t < t*_2_)

*S*_2_ is off, and the voltage $v_{DS2}$ is maintained at $V_{in}$. The current $i_{l2}$ converts from *S*_2_ to flow through *D*_2_ to the output terminal. From Figure 4b, the voltage equations of the buck converters are as follows:

(7) $$v_{l1}+v_{T}+V_{LED}=V_{in}$$

(8) $$v_{l2}+v_{T}+V_{LED}=0$$

Adding (7) and (8) results in:

(9) $$V_{in}=\left(v_{l1}+v_{l2}\right)+2v_{T}+2V_{LED}$$

Applying Faraday’s law to *L_l_*_1_ and *L_l_*_2_ and using (3) results in:

(10) $$v_{l1}+v_{l2}=\frac{L_{l}di_{l1}}{dt}+\frac{L_{l}di_{l2}}{dt}=\frac{L_{l}di_{lm}}{dt}$$

Substituting (4) and (10) into (9) yields:

(11) $$v_{T}=\frac{L_{M}}{L_{l}+2L_{M}}\left(V_{in}-2V_{LED}\right)$$

Substituting (11) into (7) and (8) yields:

(12) $$v_{l1}=\frac{L_{M}+L_{l}}{L_{l}+2L_{M}}V_{in}-\frac{L_{l}}{L_{l}+2L_{M}}V_{LED}$$

(13) $$v_{l2}=-\frac{L_{M}}{L_{l}+2L_{M}}V_{in}-\frac{L_{l}}{L_{l}+2L_{M}}V_{LED}$$

Because *L_M_* is much larger than *L_l_*, (12) and (13) show that $v_{l1}$ is positive and $v_{l2}$ is negative. Therefore, $i_{l1}$ rises and $i_{l1}$ decreases. In other words, when *S*_2_ is turned off, the current flowing through the secondary winding of the coupled inductor is converted to flow through the primary winding. When the current $i_{l1}$ drops to zero, the circuit enters the next operating mode.

##### Mode III (*t*_2_ *< t < t*_3_)

This mode describes the short duration of discharging *C_DS_*_2_ to −0.7 V. From Figure 4c, the voltages across *L_l_*_1_ and *L_l_*_2_ are:

(14) $$v_{l1}=V_{in}-v_{T}-V_{LED}$$

(15) $$v_{l2}=V_{in}-v_{T}-v_{DS2}-V_{LED}$$

Adding (14) and (15) yields:

(16) $$v_{l1}+v_{l2}=2V_{in}-2v_{T}-2V_{LED}-v_{DS2}$$

Substituting (4) and (10) into (16) derives:

(17) $$v_{T}=\frac{L_{M}}{L_{l}+2L_{M}}(2V_{in}-2V_{\mathrm{LED}}-v_{\mathrm{DS}2})$$

Substituting (17) into (14) and (15) yields:

(18) $$v_{l1}=\frac{L_{l}}{L_{l}+2L_{M}}\left(V_{in}-V_{LED}\right)+\frac{L_{M}}{L_{l}+2L_{M}}v_{DS2}$$

(19) $$v_{l2}=\frac{L_{l}}{L_{l}+2L_{M}}\left(V_{in}-V_{LED}\right)-\frac{L_{l}+L_{M}}{L_{l}+2L_{M}}v_{DS2}$$

At the beginning of this mode, $v_{DS2}$ is equal to *V_in_*. Equation (19) reveals that $v_{l2}$ is negative. Therefore, $i_{l2}$ continues to decrease from zero to become negative. The capacitance $C_{DS2}$ is discharged, and $v_{DS2}$ decreases. From (17) to (19), it can be seen that *v_T_* and $v_{l2}$ increase, while $v_{l1}$ decreases. As soon as $v_{DS2}$ decreases to −0.7 V, *D_S_*_2_ is turned on, and the circuit begins operating in mode IV.

##### Mode IV (*t*_3_ *< t < t*_4_)

From Figure 4d, neglecting the diode conducting voltage, the voltage equations of the buck converters are:

(20) $$v_{l1}+v_{T}+V_{LED}=V_{in}$$

(21) $$v_{l2}+v_{T}+V_{LED}=V_{in}$$

Adding (20) and (21), we obtain:

(22) $$2V_{in}=\left(v_{l1}+v_{l2}\right)+2v_{T}+2V_{LED}$$

Substituting (4) and (10) into (22) derives:

(23) $$v_{T}=\frac{2L_{M}}{L_{l}+2L_{M}}\left(V_{in}-V_{LED}\right)$$

Substituting (23) into (20) and (21), we obtain:

(24) $$v_{l1}=v_{l2}=\frac{L_{l}}{L_{l}+2L_{M}}\left(V_{in}-V_{LED}\right)$$

From (23) and (24), $v_{l1}$, $v_{l2}$, and $v_{T}$ are all positive, so $i_{l1}$, $i_{l2}$, and $i_{lm}$ all increase. Because *L_M_* is much larger than *L_l_*, (24) shows that $v_{l1}$ and $v_{l2}$ are small, and $i_{l1}$ and $i_{l2}$ slowly rise. Before $i_{l2}$ rises to zero, $v_{\mathrm{GS}2}$ changes from low to high level in this mode. When $i_{l2}$ rises to zero and changes its polarity, it flows through *S*_2_, and the circuit enters the next operating mode.

##### Mode V (*t*_4_ *< t < t*_5_)

*S*_1_ and *S*_2_ are both on. Before *S*_2_ is turned on, *D_S_*_2_ is on to clamp *S*_2_ at zero volts, so *S*_2_ fulfills ZVS. Figure 4e shows the equivalent circuit of this mode. The equations of $v_{l1}$, $v_{l2}$, and $v_{T}$ are the same as those of mode IV, so $i_{l1}$, $i_{l2}$, and $i_{lm}$ continue to rise. When $v_{\mathrm{GS}1}$ changes from high to low level, *S*_1_ is turned off and the circuit enters operation mode VI.

##### Mode VI (*t*_5_ *< t < t*_6_) to Mode X (*t*_9_ *< t < t*_10_)

Figure 4f–j represents the equivalent circuits of operating modes VI to X, respectively. The circuit operation of modes VI to X is similar to that of modes I to V so is not be repeated here.

When $v_{\mathrm{GS}2}$ changes from high to low level, *S*_2_ is turned off, and the circuit enters mode I of the next high-frequency cycle.

## 3. Mathematical Equation Derivation

### 3.1. Magnetizing Current and Inductance Design Equation

According to the previous assumption that *L_M_* is large enough, the magnetizing current *I_LM_* can be regarded as a constant. In addition, *L_M_* is assumed to be much higher than *L_l_*. Therefore, in mode II, from (12) and (13), $v_{l1}$ and $v_{l2}$ are approximately equal to:

(25) $$v_{l1}\approx \frac{V_{in}}{2}$$

(26) $$v_{l2}\approx \frac{-V_{in}}{2}$$

Mode III describes the process in which the parasitic capacitance of *S*_2_ is discharged from *V_in_* to −0.7 V. In general, *C_DS_*_2_ is small, so the duration of mode III is very short. During this brief period, the drop of $i_{l2}$ is also very small, so when mode III ends, $i_{l2}$ is almost equal to zero (as shown in Figure 5). 

In modes IV and V, from (24), *v_l_*_1_ and *v_l_*_2_ are approximately equal to zero:

(27) $$v_{l1}=v_{l2}=\frac{L_{l}}{L_{l}+2L_{M}}\left(V_{in}-V_{LED}\right)\approx 0$$

Because $v_{l1}$ and $v_{l2}$ are almost zero, $i_{l1}$ and $i_{l2}$ hardly change during modes IV and V. To summarize the above description, in mode II, the increasing value of $i_{l1}$ is equal to the decreasing value of $i_{l2}$, and the increasing value (or decreasing value) is approximately equal to *I_LM_*, which can be expressed as:

(28) $$I_{LM}=\frac{V_{in}}{2L_{l}}\cdot t_{f}$$

where $t_{f}$ represents the duration of mode II ($t_{f}$ = $t_{2}$ − $t_{1}$)

In mode II, $v_{T}$ is negative and $i_{lm}$ decreases. On the contrary, in modes IV and V, $v_{T}$ is positive and $i_{lm}$ increases. The sum of the durations of modes IV and V is defined as *t_r_* (*t_r_* = *t*_5_ − *t*_3_). At a steady-state operation, the average voltage of $v_{T}$ is equal to zero. Using (11) and (23) yields:

(29) $$\bar{v_{T}}=\frac{L_{M}}{L_{l}+2L_{M}}\left(V_{in}-2V_{LED}\right)\cdot t_{f}+\frac{2L_{M}}{L_{l}+2L_{M}}\left(V_{in}-V_{LED}\right)\cdot t_{r}=0$$

The sum of the fall time and rise time of $i_{lm}$ equals half of the switching period:

(30) $$t_{f}+t_{r}=0.5T_{s}$$

where *T_s_* is the switching period of the active switch. Substituting (30) into (29) gives:

(31) $$t_{f}=\left(1-\frac{V_{LED}}{V_{in}}\right)T_{s}$$

(32) $$t_{r}=\left(\frac{V_{LED}}{V_{in}}-\frac{1}{2}\right)T_{s}$$

Because both *t_r_* and *t_f_* must be positive values, (30) and (31) imply that the constraints for realizing the LED driver in this study are:

(33) $$\frac{1}{2}V_{in}<V_{LED}<V_{in}$$

As mentioned above, because the rise amount of *i_l_*_1_ in mode II is almost equal to *I_LM_*, the following equation can be deduced by substituting (31) into (28):

(34) $$I_{LM}=\frac{\left(V_{in}-V_{LED}\right)T_{s}}{2L_{l}}$$

At steady-state operation, *I_LM_* is equal to the LED current:

(35) $$I_{LED}=\frac{V_{LED}}{R_{LED}}=I_{LM}=\frac{\left(V_{in}-V_{LED}\right)T_{s}}{2L_{l}}$$

Using (35), it can be deduced that the inductance of *L_l_* is equal to:

(36) $$L_{l}=\frac{R_{LED}(V_{in}-V_{LED})}{2V_{LED}f_{s}}=\frac{(V_{in}-V_{LED})V_{LED}}{2P_{o}f_{s}}$$

where *P_o_* represents the output power of the LED driver.

### 3.2. LED Voltage Ripple Factor

**Figure 6:**
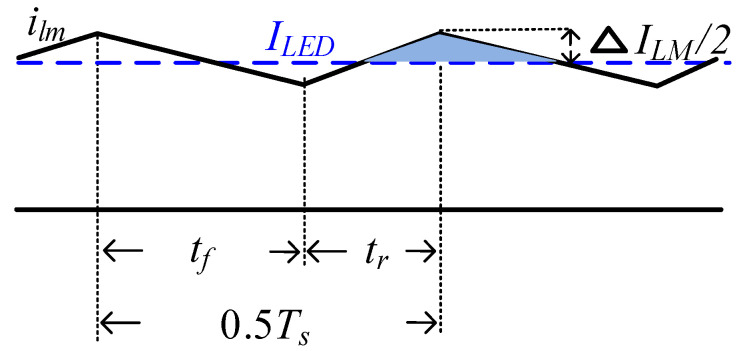
Conceptual waveform of the magnetizing current.

Figure 6 shows the conceptual waveform of the magnetizing current. Using (23) and (32), the change in $i_{lm}$ is approximately equal to:

(37) $$\Delta I_{LM}=\frac{v_{T}}{L_{M}}\cdot t_{r}\approx \frac{1}{2L_{M}f_{s}}\left(V_{in}-V_{LED}\right)\cdot \left(\frac{2V_{LED}}{V_{in}}-1\right)$$

The output capacitor is charged when $i_{lm}$ is higher than *I_LED_*. The light blue area represents the amount of charge flowing into *C_o_*, expressed as:

(38) $$\Delta Q=\frac{\Delta I_{LM}}{4}\cdot \frac{T_{s}}{2}=\frac{\left(V_{in}-V_{LED}\right)\cdot \left(\frac{2V_{LED}}{V_{in}}-1\right)}{16L_{M}f_{s}^{2}}$$

Using (38), the ripple and ripple factor of the output voltage are expressed as:

(39) $$\Delta V_{LED}=\frac{\left(V_{in}-V_{LED}\right)\cdot \left(\frac{2V_{LED}}{V_{in}}-1\right)}{16L_{M}f_{s}^{2}C_{o}}$$

(40) $$r_{v}=\frac{\Delta V_{LED}}{V_{LED}}=\frac{\left(\frac{V_{in}}{V_{LED}}-1\right)\cdot \left(\frac{2V_{LED}}{V_{in}}-1\right)}{16L_{M}f_{s}^{2}C_{o}}$$

## 4. Prototype LED Driver and Experimental Results

### 4.1. Parameter Design and Control Circuit

A 180 W prototype of the proposed LED driver was built and tested to verify its feasibility. The specifications is shown in Table 1. The load consisted of sixty 3 W LEDs, and the rated voltage and current of each LED were 3.6 V and 0.83 A, respectively. The LEDs were connected as follows: 6 strings of LEDs in parallel, with each string containing 10 LEDs in series. Hence, the output voltage and current at the rated power operation were 36 V and 5.0 A, respectively. The LED equivalent resistance *R_LED_* was calculated to be 7.2 Ω. In this illustrative example, the ripple factor of output voltage was designed to be less than 1%. 

Using (36), the values of *L_l_*_1_ and *L_l_*_2_ were obtained:

(41) $$L_{l1}=L_{l2}=\frac{\left(60-36\right)\times 36}{2\times 180\times 50\times 10^{3}}=48\;\mu H$$

For a buck converter operating in DCM, the input power is inversely proportional to the inductance value. Here, assuming 95% energy conversion efficiency, *L_l_*_1_ and *L_l_*_2_ were designed to be 45.6 μF. As mentioned earlier in this article, the magnetizing inductance should be much higher than the value of *L_l_*_1_ and *L_l_*_2_. Here, the magnetizing inductance was 732 μF, which was about 16 times that of *L_l_*_1_ and *L_l_*_2_. 

Using (40), the value of *C_o_* was calculated at a 1% ripple factor:

(42) $$C_{o}=\frac{\left(60/36-1\right)\cdot \left(2\times 36/60-1\right)}{16\times 732\times 10^{-6}\times {\left(50\times 10^{3}\right)}^{2}\times 0.01}=0.46\;\mu F$$

In order to have a ripple factor of less than 1%, a metal-film capacitor with capacitance of 1 μF was chosen. The component parameters are listed in Table 2. 

**Figure 7:**
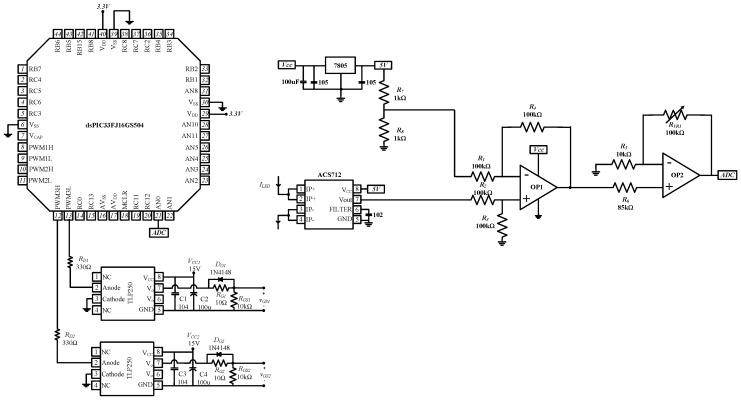
Control circuit.

As shown in (36), the output power is inversely proportional to the switching frequency. Therefore, the LED current can be regulated by a frequency control scheme. The control circuit is shown in Figure 7. The output voltages of the microcontroller (dsPIC33FJ16GS504) were fed to two gate drivers (TLP250) that output *v_GS_*_1_ and *v_GS_*_2_ to alternately turn *S*_1_ and *S*_2_ on and off. The current sensor (ACS712) sensed the LED current and output a voltage value proportional to the magnitude of the current. The difference between the output voltage of the current sensor and a reference value was measured by the operational amplifier (OP1) and sent to the other operational amplifier (OP2) for voltage amplification; then, the amplified voltage was sent to the microprocessor to adjust the switching frequency.

### 4.2. Experimental Results

**Figure 8:**
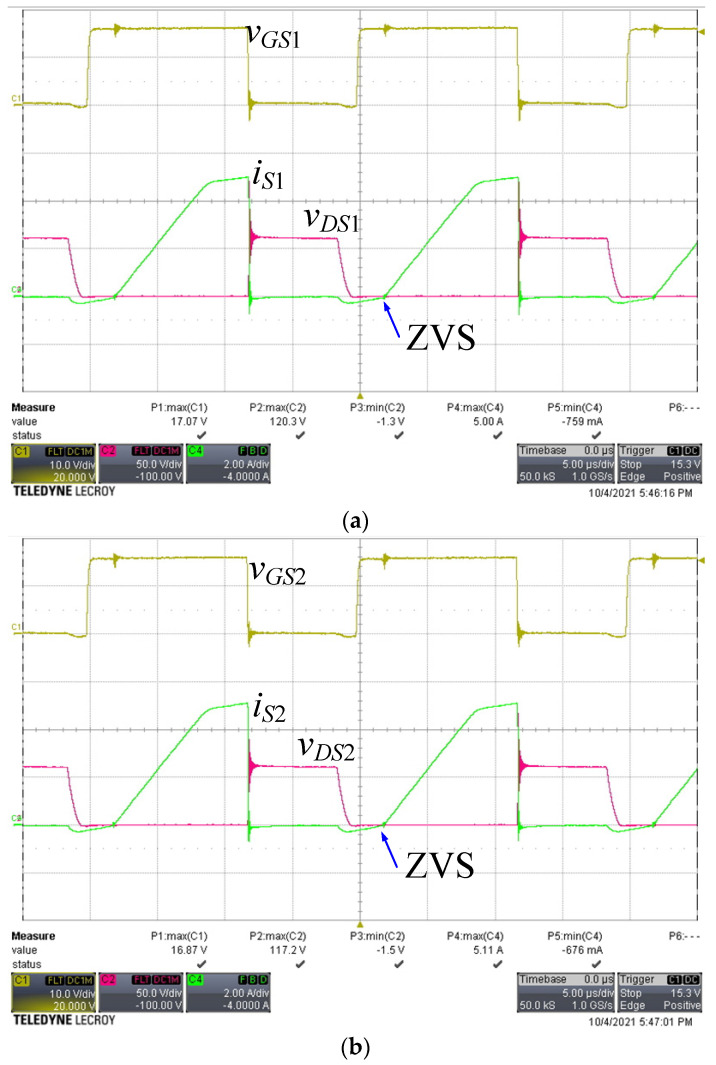
Voltage and current waveforms of the active switches. (**a**) *v_GS_*_1_, *v_DS_*_1_, and *i_S_*_1_; (**b**) *v_GS_*_2_, *v_DS_*_2,_ and *i_S_*_2_ (*v_GS_*_1_, *v_GS_*_2_: 10 V/div, *v_DS_*_1_, *v_DS_*_2_: 50 V/div, *i_S_*_1_, *i_S_*_2_: 2 A/div, time: 5 μs/div).

**Figure 9:**
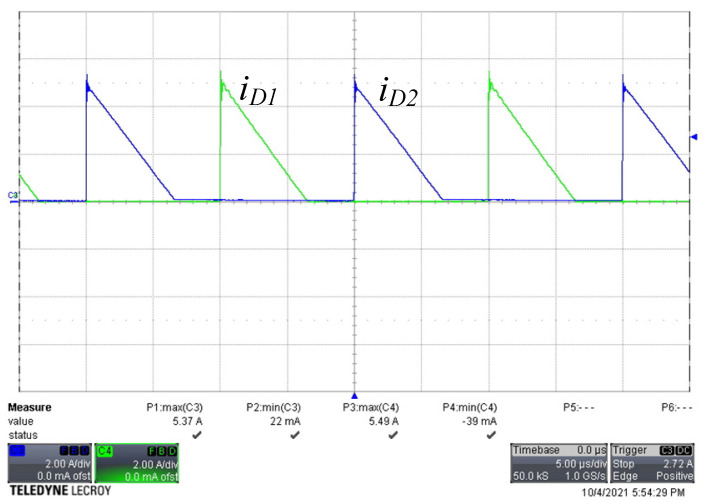
Current waveforms of the freewheel diodes. (*i_D_*_1_, *i_D_*_2_: 2 A/div, time: 5 µs/div).

**Figure 10:**
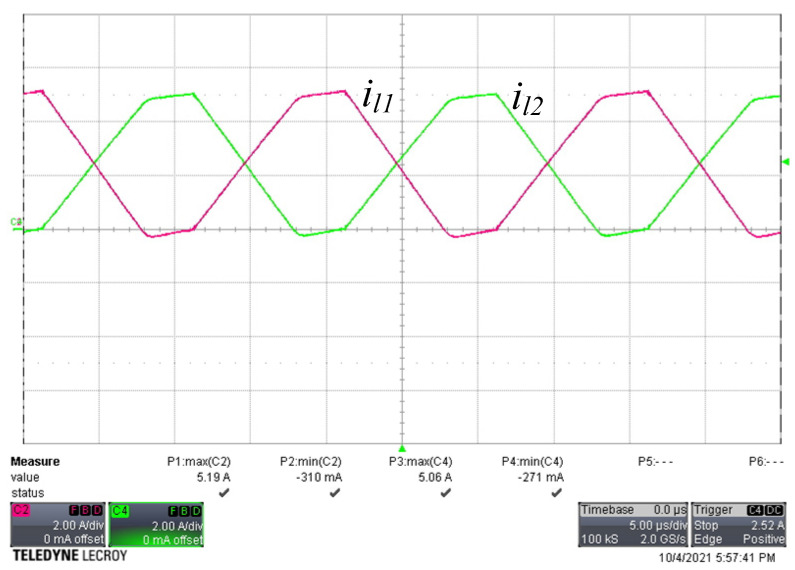
Current waveforms of the winding current of the coupled inductor (*i_l_*_1_, *i_l_*_2_: 2A/div, time: 5 µs/div).

**Figure 11:**
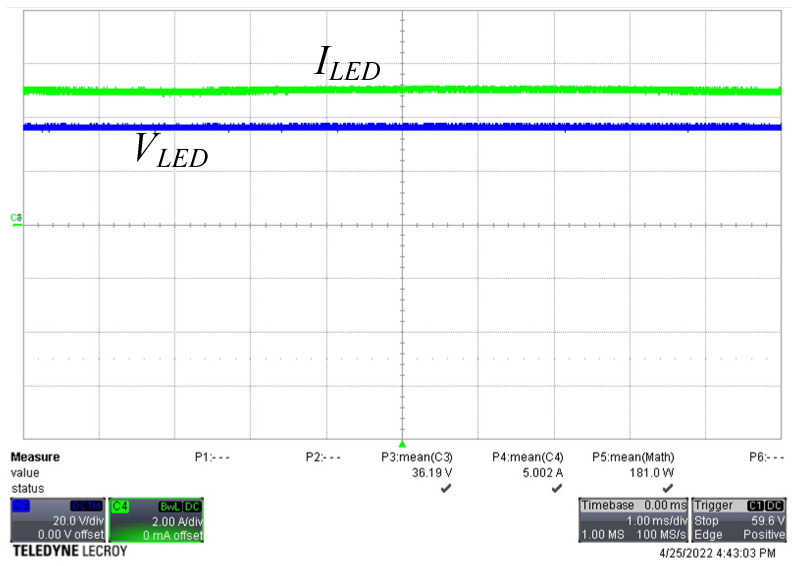
Waveforms of LED voltage and current (*V_LED_*: 20 V/div, *I_LED_*: 2 A/div, time: 1 ms/div).

Figure 8 shows the voltage and current waveforms of the two active switches. There was a small amount of negative current flowing through each active switch, and the gate voltage changed from low to high before the negative current became positive. When the negative current discharged the parasitic capacitance of the switch to −0.7 V, it turned on the intrinsic diode. Because the intrinsic diode was turned on, the voltage across the switch was clamped to almost zero voltage; therefore, the switch satisfied ZVS operation. Figure 9 shows the current waveforms of the freewheel diodes. Both diode currents dropped to zero and naturally turned off, leading to ZCS operation. Figure 10 shows the waveforms of the current in both windings of the coupled inductor. As predicted, the magnetizing current was converted between the two windings, and both winding currents dropped to slightly below zero. Figure 11 shows the output voltage and output current waveforms. The measured output power was equal to 181.2 W. The calculated ripple factor was 0.8%. The power losses in the components at the rated power operation were measured and are shown in Table 3. Due to ZVS and ZCS operation, the circuit efficiency was as high as 97.5%.

**Figure 12:**
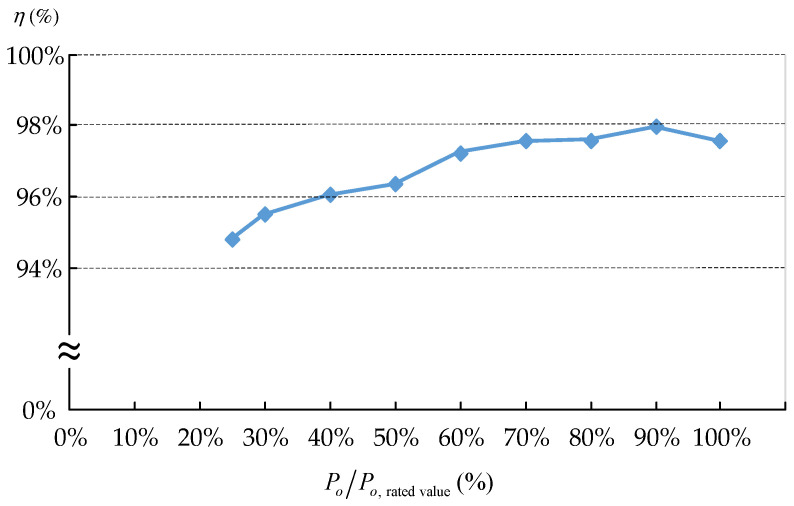
Measured circuit efficiency at different output power levels.

The LED power was controlled by adjusting the switching frequency. The measured efficiency curves over a load range of 25% to 100% rated power are shown in Figure 12. Efficiency slightly dropped when the LEDs were dimmed from 80% to 25% of the rated power.

## 5. Conclusions

In this study, an interleaved DC-DC converter was designed for driving high-power LEDs. The circuit topology consists of two buck converters, which are alternately turned on and off. A coupled inductor consisting of a magnetic core and two windings is shared by the two buck converters. To maintain the magnetic flux continuity, the magnetizing current is converted between the two windings. In this way, the freewheel diodes can operate at ZCS and the active switches can operate at ZVS. In addition, because the magnetizing current is nearly constant and the buck converter continuously supplies the magnetizing current to the output terminal, the ripple voltage remains very small even when a small metal-film capacitor is used.

Detailed steady-state operation was analyzed and the mathematical equations for different modes were derived; thereafter, the component parameters were designed. Finally, a 180 W prototype circuit was implemented to drive sixty 3 W LEDs. By adjusting the switching frequency of the active switches, the LED power was dimmed from 100% to 25% rated power. Owing to ZVS and ZCS operation, the switching losses of the semiconductor devices were effectively reduced, leading to a high circuit efficiency. The measured circuit efficiency was as high as 97.5% at the rated power operation. At a low power operation, the circuit efficiency remained high. When the circuit was operated at 25% of its rated power, the efficiency was 94.7%. The output capacitor was a 1 μF metal-film capacitor; the measured ripple factor of the output voltage was as low as 0.8%. Without the use of any bulk electrolytic capacitors, the long lifetime of the proposed LED driver can be ensured. The experimental results verified the feasibility of the proposed circuit.

## Figures and Tables

**Table 1:** Specifications of the proposed LED driver.

Item	Value
Input Voltage, *V_in_*	60 V
Output Voltage, *V_LED_*	36 V
Output Power, *P_o_*	180 W
Voltage Ripple Factor, *r_v_*	<1%
Switching Frequency, *f*_s_ (at rated power)	50 kHz
3 W LED Rated Voltage	3.6 V
3 W LED Rated Current	0.83 A

**Table 2:** Component parameters.

Item	Value
Inductance, *L_l_*_1_, *L_l_*_2_	45.6 μH, 45.6 μH
Magnetizing Inductance, *L_M_*	732 μH
Output capacitance, *C_o_*	1 μF (metal-film capacitor)
Diodes *D*_1_, *D*_2_	C3D10060A
Active switches *S*_1_, *S*_2_	STW52NK25Z

**Table 3:** Measured losses in the components.

Item	Value
Coupled Inductors, *L_M_*	0.8 W
Inductors, *L*_1_, *L*_2_	0.23 W, 0.23 W
Active Switches, *S*_1_, *S*_2_	0.8 W, 0.8 W
Diodes, *D*_1_, *D*_2_	0.7 W, 0.7 W
Output Capacitor, *C_o_*	0.3 W
